# Preparation and Characterization of Cyanocobalamin (Vit B_12_) Microemulsion Properties and Structure for Topical and Transdermal Application

**Published:** 2013-07

**Authors:** Anayatollah Salimi, Behzad Sharif Makhmal Zadeh, Eskandar Moghimipour

**Affiliations:** 1 Nanotechnology Research Center, Ahvaz Jundishapur University of Medical Sciences, Ahvaz, Iran; 2 School of Pharmacy, Ahvaz Jundishapur University of Medical Sciences, Ahvaz, Iran

**Keywords:** DSC, Microemulsion microstr- ucture, Pseudo ternary phase- diagram, SAXS

## Abstract

***Objective(s):*** The objective of this study was to design a topical microemulsion of Vit B_12 _and to study the correlation between internal structure and physicochemical properties of the microemulsions. Microemulsions are thermodynamically stable mixtures of water, oil, surfactants and usually cosurfactants with several advantages for topical and transdermal drug delivery. The formulation of microemulsions for pharmaceutical use requires a clear understanding of the properties and microstructures of the microemulsions.

***Materials and Methods:*** In this study, phase behavior and microstructure of traditional and novel microemulsions of Vit B_12 _have been investigated by Small-angle X-ray (SAXS), differential scanning calorimetery (DSC) and measuring density, particle size, conductivity and surface tension.

***Results:*** WO and bicontinuous microemulsion with different microstructures were found in novel and traditional formulations. In this study, amount of water, surfactant concentration, oil/ surfactant ratio and physicochemical properties of cosurfactants influenced the microstructures. In both formulations, water behavior was affected by the concentration of the surfactant. Water Solubilization capacity and enthalpy of exothermic peak of interfacial and free water of traditional formulations were more than novel ones. This means that the affinity of water to interfacial film is dependent on the surfactant properties.

***Conclusion:*** This study showed that both microemulsions provided good solubility of Vit B_12 _with a wide range of internal structure. Low water solubilization capacity is a common property of microemulsions that can affect drug release and permeability through the skin.  Based on Vit B_12 _properties, specially, intermediate oil and water solubility, better drug partitioning into the skin may be obtained by traditional formulations with wide range of structure and high amount of free and bounded water.

## Introduction

Microemulsions are thermodynamically stable and low viscose mixtures of oil and water that have been stabilized with a surfactant and usually in combination with a cosurfactant. Microemulsions have shown several advantages for drug delivery such as; ease of preparation, perfect stability, increasing drug solubility, controlling drug delivery rate, improving bioavailability of hydrophilic and lipophilic drug through different delivery routes (1, 2). Advantages of microemulsions in topical and transdermal drug delivery have been suggested by several studies. The perfect drug delivery properties of microemulsions are attributed to the solubility properties and their penetration enhancement effects. Although, the correlation between microemulsion structure and composition and successful topical and transdermal drug delivery is not fully explained but a few studies have presented knowledge on interaction of the inner structure of the microemulsion and drug penetration into the skin (3, 4). The mixture of water, oil, surfactant and cosurfactant may lead to 3 types of microemulsions: water in oil, oil in water and bicontinuous with a wide variety of structures (5, 6). The perfect physicochemical properties of microemulsion such as; high drug loading, drug solubilization and high fluctuating interface are better obtained in the bicontineous structures than in globular microemulsions (7, 8). On the other hand, the type of microemulsion influences drug release rate. Relatively fast drug release from bicontinuous structures occurs for both water and oil soluble drugs. For W/O and O/W microemulsions, both hydrophilic and lipophilic drugs demonstrate fast drug release. Phase behavior, microstructure and stability of microemulsions may be affected by the drug that is loaded into the microemulsion (9). Therefore, in order to determine drug delivery potential of the microemulsion, it is important to characterize the microstructure and define properties of the microemulsion. Different techniques such as differential scanning calorimetry (DSC), X-ray diffraction, particle size analyzer, conductivity, surface tension and viscosity are used for microemulsion characterization (9, 10).  

In this study, microemulsions were prepared with stearylamine and tween80/span20 as ionic and nonionic surfactants, respectively, and labrafil and propylene glycol as cosurfactant and oleic acid as oil phase. Cyanocobalamin (Vit B_12_) with molecular weight of 1354 g/mol was used as drug model and the effect of drug, surfactant and other independent variables on microemulsion phase behavior, properties and microstructures were evaluated. Vit B12 is an effective nitric oxide scavenger and so can suppress cytokine production and demonstrate anti inflammatory effects for atopic dermatitis (11). But in the case of low bioavailability of Vit B_12_, systematic administration in skin disorders such as psoriasis dose not demonstrate therapeutic effects (12).  On the other hand, effective transdermal delivery can introduce a perfect alternative delivery route to Vit B_12_ injection. So, clearly for designing a topical microemulsion of Vit B_12_ with perfect efficacy**,** sufficient information about physicochemical properties and internal structure of microemulsions is needed, which was studied in this research by different experiments such as phase diagram, internal structure evaluation by DSC and SAXS, particle size determination and viscosity.

## Materials and Methods

Vit B_12 _and labrafil were donated by Iran Hormone pharmaceutical Company (Tehran, Iran) and Gattefosse Company (France), respectively. Tween 80, span 20 and oleic acid were purchased from Merck (Germany) and sigma Aldrich respectively. Stearylamine was purchased from Fluka (Germany). All other materials were of the highest grade commercially available. 

Study design for preparation of microemulsions Several parameters influence the final properties of microemulsions. Full factorial design was used concerning 3 variables at 2 levels for two main formulations including novel and traditional formulations (Table 1). Major variables that determine microemulsion’s properties include surfactant/cosurfactant ratio (S/C) and percentages of oil (% oil) and water (%w).  According to the table, S/C ratio, % oil, % water, surfactant and co-surfactant in novel and traditional formulations were different, whereas the oily phase in both formulations was the same. Oil, surfactant and co-surfactant were selected based on their drug solubility capacity, HLB values and ability of microemulsion formation. Alcohol was incorporated into novel microemulsion systems to dissolve the drug and increase the curvature of the oil layer. In the present study, the influence of independent variables on particle size, zeta potential, conductivity, thermal behavior, X-ray diffraction pattern and microstructure of microemulsions considered as responses were investigated. The interactions intensity of variables on each response were estimated through simultaneous multiple regression.

**Table 1:** Presentation of independent variables and components of novel and traditional formulations

Parameters			Traditional formulation	Novel formulation
Variables	S/C	+	3/1	1/8
		-	1/1	1/4
	% 0il	+	50	70
		-	5	30
	% w	+	10	20
		-	5	5
surfactant		Tween80+span20 (1:1)		Stearylamine
Cosurfactant		PG		Labrafil/ alcohol
Oily phase		Oleic acid		Oleic acid

High level= +, low level=-), S/C= surfactant/ cosurfactant ratio, w=water, PG=propylene glycol

***Screening of oils for microemulsions***

Drug solubility in oil phase is a critical parameter for finding the suitable oil that can be used as oil phase. The solubility of Vit B_12_ in mineral oil (MO), isopropyl myristate (IPM), oleic acid (OA) and Labrafil M 1944 (LAB) were measured. Then**, **the solubility of Vit B_12_ in the mixtures of oils and Transcutol p (10:1) as solubilizer was evaluated. For this purpose, an excess amount of drug was added to oily and mixtures of oil and Transcutol, stirred for 48 h at room temperature and centrifuged for 20 min at 10000 rpm to remove undissolved Vit B_12_. In the next step, the concentration of Vit B_12 _was measured with UV method at 362 nm. 

***Microemulsion preparation and phase diagram***

To investigate the microemulsion formation region, microemulsions were prepared using titration method. For this purpose, phase diagrams were constructed at room temperature by admixing defined quantities of components without drug (as control) or in the presence of 0.07% of Vit B_12_. Using this method preparation was performed over 3 phases. In the first phase, stock solution of surfactant and cosurfactant was prepared and different amounts of oily phase were added and then titrated with water and stirred. In the second phase, different ratios of mixtures of surfactant, cosurfactant and water were well stirred and then the oily phase was titrated. In the third phase, different ratios of oily phase and water were prepared and titrated with different amounts of surfactant and cosurfactant mixture. Finally, compositions of titrated samples were calculated and plotted in triangular co-ordinates to construct the pseudoternary phase diagram (13, 14).

***Particle size and zeta potential determination***

Droplet size and polydispersity index (PDI) of both novel and traditional formulations were measured by application of dynamic light scattering (Malvern, master sizer SM 2000k, United Kingdom) at room temperature using neon laser at 632 nm. The measurements were triplicated for checking reproducibility. 

***Conductivity measurements***

Electric conductivities of both formulations were measured by mettler Toledo 226 (Switzerland) conductivity meter with constant of 0.726 cm^-1^. Measurements were made in triplicate at 20ºC. 

***Differential scanning calorimetry (DSC)***

DSC measurements were carried out by a Mettler Toledo DSC star system equipped with refrigerated cooling system (Hubert Tc45). Approximately, 10-15 mg of each microemulsion sample was weighted into hermetic aluminum pans and quickly sealed to prevent water evaporation. Simultaneously**,** an empty hermetically sealed pan was used as a reference. Microemulsion samples were exposed to heat ranging from 25^0^C to 200^0^C (scan rate:10 ^0^C/min). In the same procedure, samples were kept in temperature ranging from 30^0^C to - 50^0^C (scan rate: 100^0^C/min).  All experiments were done at least in triplicate. In order to ensure accuracy and repeatability of data, DSC analyzer was calibrated and checked under the experiment conditions by indium standard. Enthalpy (∆H) quantities were calculated from endothermic and exothermic transitions of thermograms by Eq1 (5):

∆H= transition peak area/sample weight………………….(1)

***X-ray scattering***

  X-ray diffraction (XRD) characterization of microemulsions were carried out using a Philips PC-APD diffractometer (XPert MPD) with Goniometer type PW3050 /e-2e Ni-filtered Co Kα radiation (λ=1.78897 ^0^A) at operating power generator 40KeVand 30MA was used. θ-2θ scans were made for all microemulsion samples. The ranges of XRD measurements were usually from 1.11 to 9.99^0^2θ with scanning rate of 0.02^0^/sec. The samples were transferred to a spinner stage in a thermally controlled sample holder centered in the X- radiation beam. The scattering intensities data were collected at MINIPROP detector.  All X-ray scatterings were done at 25^0^C (10).  X-ray diffraction was conducted at X-ray laboratory at Tarbiat Modares University, Tehran, Iran.

***Morphology characterization***

Transmission electron microscopy (TEM) was applied to determine the microstructure of microemulsions. The TEM images were provided by Tecnai G2 20 (Germany) and samples were placed on a carbon coated copper grid. 

***Stability of microemulsions***

Five milliliter of drug-loaded microemulsion samples was stored at three temperatures (4, 25 and 40ºC) for 2 months. During this period, samples were checked for any turbidity and coalescence. In addition, samples were centrifuged at 10000 rpm for 2 months for determination of physical instability such as phase separation and aggregation (15).

***Statistical analysis***

Data is demonstrated as mean ± S.D. The statistical analysis was based on un-paired t-test or variance analysis, followed by full-factorial design using Minitab 11 software. In order to find out the relation between dependent and independent variables, multi regression test was applied simultaneously.

## Results

***Validity of drug measurement method***

Drug assay was performed by spectrophotometer at 362 nm. The relationship between the light absorption and concentration was significant (R^2^ = 0.993, *P*<0.002). A repeated survey accountability in measurement method within and between days demonstrated perfect repeatability. The difference between real numbers and the estimated correlation that represents accuracy was about 6% which indicates the closeness of the estimated values and actual results. 

***Solubility of Vit B***_12_ ***in various oils***

Solubility of Vit B_12_ in various oily phase and mixtures of oil and Transcutol as solubilizer at room temperature are shown in Table 2. The solubility of Vit B_12_ in oleic acid was significantly (*P*<0.05) greater than other oils, so it was chosen as oil phase. Transcutol increased drug solubility in different oily phases significantly (*P*<0.05). Therefore, oleic acid and Transcutol (10:1) were selected as oily phase for both formulations.

**Table 2:** Vit B_12_ in different oil phase with and without transcutol (mean ± SD, n=3)

Oil	Solubility (mg/ml)	
	Without transcutol	With transcutol
Oleic acid	1.07 ± 0.3	7.53 ± 0. 2
Isopropyl myristate	0.42 ± 0.002	0.98 ± 0.006
Labrafil	0.048 ± 0.001	0.11 ± 0.003
Mineral oil	0.026 ± 0.0001	0.052 ± 0.002

***Development of microemulsions and Phase behavior***

**Figure 1:**
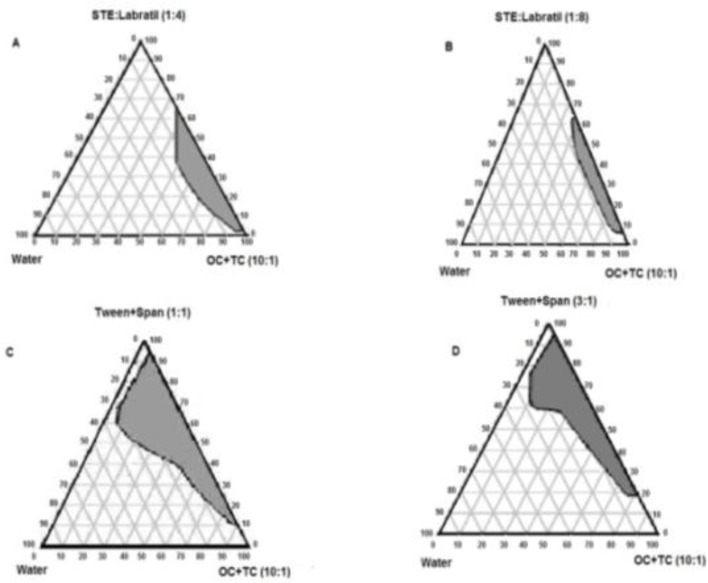
Phase diagrams of novel (A, B) and traditional(C, D) formulations with different ratios of surfactant/cosurfactant

The behavior of four-component microemulsion was demonstrated with pseudoternary phase diagrams (Figure 1) for traditional (A) and novel (B) formulations. 

***Microemulsion properties***

Particle size, pH, Zeta potential, conductivity and surface tension of microemulsions are shown in table 3. Particle size of traditional and novel formulations was between 49-209 and 17-210 nm**,** respectively, with a polydispersity index of below 0.5 for both formulations. The interfacial tension of both formulations was formed in the range of 3.9-5 mN/m. 

***DSC***

Cooling microemulsion’s transition temperature and enthalpy are provided in (Table 4).  

***Small-angle X-ray Scattering***

**Figure 2:**
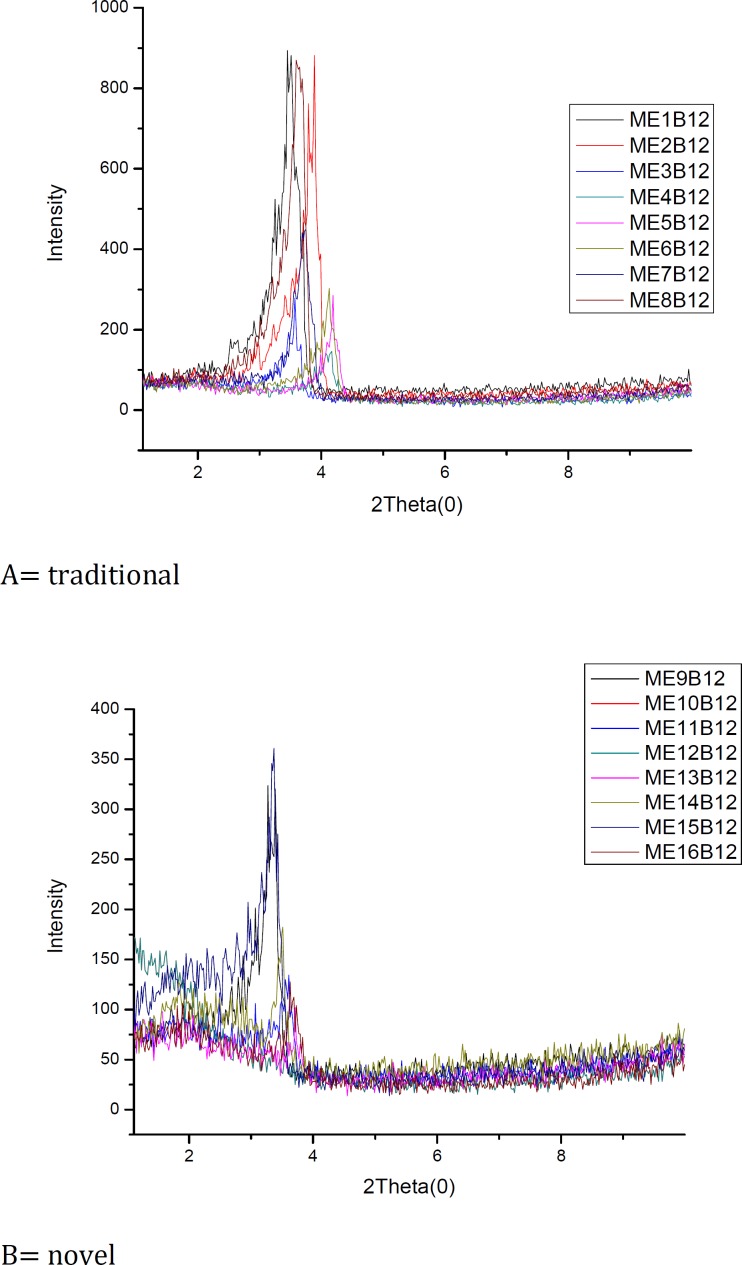
Small angle X-ray scattering curves for (A) traditional and (B) novel microemulsions

In this study, Small-angle X-ray Scattering was applied to study the traditional and novel microemulsions. The results of SAXS experiments on the different microemulsion formulations are shown in Figure 2. 

## Discussion

Comparison of phase diagrams of novel and traditional formulations demonstrated two main results; -traditional formulation with nonionic surfactant indicated more water solubility capacity than novel formulations with cationic surfactant. Higher microemulsion regions were shown with higher concentration of cosurfactant. In both formulations**,** oil phase was the same and cosurfactants were different. Microemulsion regions were obtained with 20-90 and 5-70% of surfactant and cosurfactant for traditional and novel formulations, respectively. Therefore**,** higher amounts of surfactant and cosurfactant are necessary for traditional microemulsion formation. 

**Figure 3:**
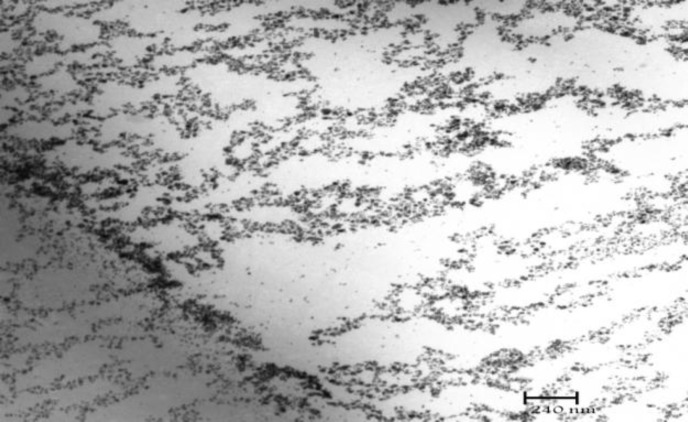
TEM image of microemulsion no.5 with bicontinuous structure

**Table 3:** pH, particle size, zeta potential, conductivity and surface tension of selected microemulsions (mean±SD, n=3)

Formulation	Factorial design condition	pH	Mean of particle size ± SD	Zeta potential (mv) ± SD	Conductivity (m.s/cm)	Surface tension (mN/m)
1	+++	4.73	89 ± 7.29	-45.9±3.3	8.4	4.2 ±7
2	++-	4.79	52 ±11.3	-54.2±6.6	7.2	4.1 ±6
3	+-+	6.42	209 ±14	-34.2±4.1	7	4.9 ±2
4	+--	6.45	199 ±16	-35.4±5.2	6.9	5.1± 4
5	--+	6.25	119 ±15	-39.2±2.5	5.9	4.9 ±5
6	---	6.35	117 ±7.1	-34.2±3.5	7.9	4.5 ±2
7	-+-	4.69	49 ±2.3	-53.7±4.7	8.5	4.62 ±2.5
8	-++	4.82	63 ±8.15	-51±7.3	7.4	4.6 ±2.8
9	+++	4.37	62± 3	-42.4±2.6	8.1	4.7± 3
10	++-	4.82	210 ±12	-43.4±5.6	8	4.29 ±3
11	+--	5.93	205 ±14	-23.6±4.1	7.5	4.8 ±2.5
12	+-+	5.52	203± 10	-24±1.3	6.9	5.0 ±1.5
13	--+	5.57	36 ±2	-33.5±2.7	6.2	3.9 ±3
14	---	5.37	30± 4	-29.5±1.8	9	4.1± 6
15	-+-	4.42	29± 3	-26±4.1	10	4.25 ±4
16	-++	4.50	17 ±2.5	-28.4±3.4	9.5	4.8 ±4

**Table 4:** Transition temperature and enthalpy of microemulsions

Formulation	Factorial design condition	Cooling	
		T_m_(^0^c)	∆H(J/g)
ME-1	++-	-12 -27	-32.66 -48.71
ME-2	+-+	-11 -36	-40.41 -11.55
ME-3	+--	-24 -9.3	-30.43 -63.41
ME-4	--+	-4.5 -9.4 -24	-38.14 -1.23 -2.43
ME-5	---	-4 -9	-13.48 -7.01
ME-6	-+-	-4.5 -12 -40	-16.91 -4.41 -2.63
ME-7	-++	-4 -31	-28.58 -22.66
ME-8	+++	-9.8 -36	-64.69 -3.79
ME-9	++-	-11.9 -35	8.89 37.88
ME-10	+--	-11.6 -24	7.87 35.77
ME-11	+-+	-9.8 -24	4.5 0.57
ME-12	--+	-4.5 -44.5	2.29 -2.43
ME-13	---	-2	2.6
ME-14	-+-	-4 -44.4	2.71 20.63
ME-15	-++	-5 -30	6.71 27.04
ME-16	+++	-9 -31.5	7.7 33.04

 It seems that phase behavior depends on surfactant and cosurfactant properties. Propylene glycol and labrafil/ethanol were used as cosurfactant in traditional and novel formulsions, respectively. Higher efficacy of propylene glycol as hydrophilic cosurfactant to improve emulsification of surfactant than labrafil as hydrophilic cosurfactant was reported (16). On the other hand**,** labrafil with oleate backbone demonstrated lower penetration into interfacial surfactant layer than propylene glycol with short chain and low molecular volume that provided good interfacial fluidity (17). Therefore, cosurfactant properties (such as molecular volume and HLB) influence on microemulsion phase behavior and propylene glycol with higher hydrophilicity and lower molecular volume demonstrated higher microemulsion region and water solubility. The weight ratio of surfactant/cosurfactant is a critical and important parameter affecting phase behaviors of microemulsion. Increase in microemulsion region with higher concentration of surfactant was reported (17). Findings in the present study indicate an increase in microemulsion region for traditional and novel formulations at higher cosurfactant concentration. At room temperature, water is a good solvent for the non-ionic surfactant and at higher temperatures the surfactant becomes soluble in oil. At room temperature, a surfactant-rich water phase with an oil-excess phase and at high temperatures, a coexistence of surfactant-rich oil phase with a water-excess phase was found (18, 19). Our results indicate that non ionic surfactant provided larger microemulsion region and soluble oil more than water. These findings suggest that cosurfactant increased the solubility of oil in room temperature non-ionic surfactant. On the other hand**, **decrease in cosurfactant in surfactant-cosurfactant mixtures, decreases oil solubility.  

All ionic surfactants with one single hydrocarbon chain are too hydrophilic to make up microemulsions. With these ionic surfactants, microemulsion can only be formed by adding electrolytes or cosurfactants. The phase behavior of pseudoternary ionic surfactants can be described with three binary systems. At room temperature, ionic surfactants are soluble in oil phase and at high temperatures in aqueous phase (20). In the present study, ionic surfactant same as- non ionic surfactant in the mixture with cosurfactant soluble oil more than water at room temperature. Comparison between ionic and non-ionic surfactants used in this study indicates that:

 - Water solubility of non-ionic surfactants were more than ionic surfactants. This behavior is dependent on cosurfactant concentration. Water solubility increased at higher concentration of co-surfactant. Higher surfactant-cosurfactant concentration is needed for microemulsion formation in non-ionic surfactant. 

Novel formulations demonstrated lower mean particle size. Correlation between particle size and S/C ratio was direct and significant for both formulations (*P*<0.05). It was indirect and significant for oil% (*P*=0.015) in traditional and not significant for novel formulations. These results suggest that increase in the concentration of cosurfactant makes a significant decrease in the mean particle size that reflects the role of cosurfactants on producing efficient curvature and then reducing particle size that has been reported (21). It seems that the effect of oil on particle size depends on properties of surfactant and cosurfactant. In traditional formulations, the droplet size was found to increase with increasing oil percentage and it is evident that 50% w/w of oleic acid is the optimum oil concentration that can perform microemulsion with perfect particle size. 

The relation between S/C ratio (*P*=0.05) and oil% (*P*=0.005) with surface tension was indirect and significant in traditional formulations and for novel formulations no significant relation was found. 

The effect of oil% on surface tension and particle size in traditional formulation were similar. This finding suggests that increase in oil% by reducing interfacial tension and particle size induced winsor Winsor system change from o/w to w/o followed by reduction in interfacial tension and particle size. On the other hand**,** the effect of S/C on particle size was opposite of surface tension. 

Electrical conductometry is a useful tool for evaluation of conductive behavior of microemulsion samples. Due to the conductivity properties of aqueous phase, o/w microemulsions demonstrate higher conductivity values than the w/o microemulsions (22). Comparison between conductivity of microemulsions and blank (microemulsion without Vit B _12)_ indicated that Vit B_12_ did not change the conductivity that may be due to no drug dissociation in media. On the other hand, no significant difference between the conductivity of traditional and novel formulation was found. Other independent variables didn’t indicate any significant correlation with conductivity.

Differential Scanning Calorimetry (DSC) was used to measure heat flow associated with transitions in materials as a function of temperature. DSC results provide useful information about the physical and chemical changes that involve endothermic or exothermic processes or changes in heat capacity (23). DSC study was used for water behavior in microemulsions and distinction between bulk (free) and bound (interfacial) water (23).

In the traditional formulations (No 1-8), DSC profiles showed 1 exothermic peak at around -9 to -14°C which is attributed to the bounded water that is strongly bounded to the surfactant at the interface (23). The correlation between S/L ratios with enthalpy of transition occurring at -9 to -14 (∆H_b_) was significant in such a way that increase in the independent variables lead to significant increase in the enthalpy.  Another exothermic peak appeared around -4 to -5°C in the formulations with low amount of S/L ratio, attributed to the bulk (free) water (23). It seems that at high S/L ratio all the water in the traditional microemulsions is interfacial water (bounded) because by decreasing surfactant concentration, the exothermic peak of bulk water appeared.  Similar behavior was found in novel formulations, where bounded water was observed only in formulations with low S/L ratio. Therefore**,** in both formulations**,** water behavior is affected by surfactant concentration. Water solubilization capacity, enthalpy of exothermic peak of interfacial and free water of traditional formulations is more than novel ones. This means that the affinity of water to interfacial film is dependent on surfactant properties.

SAXS techniques have been used by several researchers to gain information about droplet size and microstructure of microemulsions. The periodic spacing (d) was calculated by q (d=2π/q). The effect of Vit B_12_ and independent variables on scattering property and internal structure of the microemulsions were explored.  For traditional formulations, bicontinuous phase was detected for formulations 3-6 which include approximately equal volumes of oil and water. Bicontinuous structure is proved by TEM micrograph (Figure 3). A very sharp scattering peak which is followed by another less pronounced one was observed in formulations 3 & 4. These two peaks are equidistant and indicate lamellar structure (2). Decrease in surfactant and cosurfactant amount in formulations 5 & 6, changed lamellar structures into cubic. This effect of surfactant concentration on microstructure of microemulsion has been reported previously (19). Reverse hexagonal structure was found in formulations 2 & 8 which present the highest oil/ surfactant ratio. It seems that high oily phase/ surfactant ratio produced high-ordered structure. This finding is in conflict with a previous study that indicated destabilizing effect of oil on hexagonal structure (24). Other traditional formulations demonstrate cubic structure and sphere micelles. The effects of s/c and Vit B_12 _on microstructure were not significant. On the other hand**,** surfactant concentration, water amount and oil/ surfactant ratio showed significant effects on microstructures. High scattering pattern was found with small amount of water as previously reported (10). The scattering increases with addition of water. In novel formulations, reverse micelles with cubic structure were found in formulations 9, 10, 11, 13, 14 and 15. Bicontinuous microemulsions with lamellar structures were demonstrated in formulation 12 and w/o microemulsion with hexagonal structure in formulation 16 which has the highest amount of oil/ surfactant ratio. Comparison between microstructure of traditional and novel formulations indicates that lamellar structure was formed in traditional formulation with non-ionic surfactant more than novel formulation with ionic surfactant. The main structure in novel formulations is cubic, while for traditional formulations a wide range of structures such as lamellar, cubic and hexagonal were found. Vit B12 changed microstructures from cubic into hexagonal in formulations 11, 13 and 14. It seems that repulsion between ionic head groups in novel formulations limited the number of microstructures. Alcohol that was used as cosurfactant in traditional formulation formed complexes with stearylamine molecules via an interaction between the hydroxyl groups of the alcohol and the ionic head groups of surfactant. Perez-Cases *et al *(25) reported the interaction of ethanol and methanol at low concentration of surfactant only with water while at high surfactant concentration they formed complexes with surfactant. It seems that in the present study the concentration of surfactant was high enough to form complexes between ethanol and surfactant. Less water uptake by novel formulations may be explained by the repulsive effect of alcohol on positive head groups of stearylamine that makes surfactant molecules pack more closely and reduces water uptake. Low water solubilization capacity is common property of microemulsions that can affect drug release and permeability through skin.  Based on Vit B_12 _properties, specially intermediate oil and water solubility, better drug partitioning into the skin may be obtained by traditional formulations with a wide range of structures and high amount of free and bounded water.    

## Conclusion

Our results clearly show more water solubility capacity for traditional formulations in comparison with novel microemulsions. Alcohol in novel formulations reduced water uptake because of interaction with stearylamine. Lower particle size was obtained with Ionic surfactant and alcohol as cosurfactant in novel formulations that produced packs closely surfactant film with usually cubic structure. Non ionic surfactant and propylene glycol made different microstructures with larger particle size that indicates loose surfactant packing film. Propylene glycol in comparison with labrafil, provided better interfacial fluidity and showed better penetration into interfacial surfactant layer and therefore higher microemulsion region and water solubility. Both kinds of microemulsion showed low conductivity that indicated w/o microemulsion. The presence of ionic surfactant in novel and higher amount of water in traditional formulations make no difference in conductivity behaviour. The location of water depends on the surfactant concentration. At low amounts of surfactant, free and bounded water was found, while at high amounts of surfactant, water was mostly located in the interfacial film. Lower enthalpy of bounded water of novel formulations as compared to traditional formulations was another evidence for low water solubilization capacity in novel formulations. SAXS and TEM indicated w/o and bicontinuous microemulsions with different microstructures in both formulations. The results showed that amount of water, surfactant concentration, oil/surfactant ratio and physicochemical properties of co-surfactants influenced microstructures of microemulsion. In conclusion, both microemulsions provided good solubility of Vit B_12 _with a wide range of internal structures. 
